# Synergistic complex from plants *Solanaceae* exhibits cytotoxicity for the human hepatocellular carcinoma cell line HepG2

**DOI:** 10.1186/s12906-016-1362-z

**Published:** 2016-10-18

**Authors:** Romina Schwarzlin, Nika Pušenjak, Damjan Makuc, Mitja Križman, Irena Vovk, Janez Plavec, Urban Švajger

**Affiliations:** 1 University of Ljubljana, Medical Faculty, Vrazov trg 2, SI-1000 Ljubljana, Slovenia; 2 National Institute of Chemistry, Hajdrihova 19, SI-1000 Ljubljana, Slovenia; 3 EN-FIST Centre of Excellence, Dunajska 156, SI-1000 Ljubljana, Slovenia; 4 Centre for Development of Advanced Therapy Medicinal Products, Blood Transfusion Centre of Slovenia, Šlajmerjeva 6, SI-1000 Ljubljana, Slovenia

## Abstract

**Background:**

It had been demonstrated that sugars from various plants can act as potent agents, which induce apoptosis of cancer cells.

**Methods:**

Using HPLC, we fractionated a mixture of two plant extracts from the plant family *Solanaceae*, namely *Capsicum chinense* and the plant family *Amaryllidaceae* namely *Allium sativum*. We evaluated the effect of different fractions on apoptosis of HepG2 cell line. The most effective fraction was further studied to determine its molecular composition using mass spectrometry (MS) and NMR. We further evaluated the effect of determined molecular composition found in the selected fraction by using a mixture of commercially available substances, which were found in the fraction and tested its pro-apoptotic effect on HepG2 cells. To get some insight into potential apoptotic mechanisms we studied caspase-3 activity and mitochondrial integrity in treated cells.

**Results:**

Out of 93 fractions obtained by HPLC from the plant extract we found HPLC fraction 10 (10 min elution) was the most effective. MS and NMR studies revealed high presence of cellobiose together with vitamin C, sulphur (S) and trace amounts of selenium (Se). HPLC fraction 10 triggered apoptosis of HepG2 within 3 h in the 0.01–1.0 mg/mL concentration range. Furthermore, a mixture of pure cellobiose, vitamin C, S and Se (complex cellobiose/C/S/Se) had a very similar capacity in inducing apoptosis of HepG2 cells compared to HPLC fraction 10. Complex cellobiose/C/S/Se was capable of inducing caspase-3 activity and led to loss of mitochondrial integrity. The capacity of cellobiose alone to induce apoptosis of HepG2 was approximately 1000-fold lower compared to complex cellobiose/C/S/Se.

**Conclusion:**

In this study we present the highly synergistic effect of a unique complex consisting of cellobiose, vitamin C, sulphur and selenium on triggering the apoptosis of human hepatocellular carcinoma (HepG2) cell line.

## Background

Cancer is a major health issue of our time and is responsible for more than 10 % of deaths worldwide and more than 25 % in some countries. The number of new cases is expected to grow by 50 % over the next 20 years to reach 15 million by 2020 [1].

Due to the widespread historical account of medicinal effects of yew plant extracts, United States Department of Agriculture (USDA) together with the National Cancer Institute (NCI) started a screening process whereby Pacific yew extracts were tested against two cancer cell lines, ovarian and breast cancer [2, 3]. An important finding resulting from this screening was paclitaxel, a hydrophobic anti-neoplastic agent found in *Taxus brevifolia* which has been shown to have anticancer properties. It is currently used for the treatment of ovarian, breast and lung cancer [4].

Exploration of the rich composition of plant extracts in general and those of *Allium sativum* and *Capsicum chinense* for its antitumor activity is not modern finding, but the beneficial effects of these plants such as antimicrobial, antithrombotic, hypolipidemic, antiarthritic, hypoglycemic and antitumor are known for centuries [5, 6]. Current researches are not limited to hydrophobic anti-neoplastic agents, but continues to shed the light on other water-soluble molecules as well, particularly sugars [7]. In this context, glucose is a known inducer of apoptosis in cancer cells, acting via several mechanisms such as disturbed metabolic pathways, reactive oxygen species (ROS) and altered release of growth factors [8].

However, the effects of other sugars, such as cellobiose, on triggering apoptosis and death of cancer cells has not yet been thoroughly studied. It was shown that sugars usually need other molecules or to be chained in polymers in order to show their apoptotic effect and induction of cell death [9]. Sugars from plants can be very potent agents to induce apoptosis of cancer cells, as it was demonstrated for colon cancer cells with cellulose experiments in mice [10]. The importance of sugars has also been demonstrated using synergistic effects of different molecules, with the demonstration that sugars and their combination with other molecules can further augment cancer cell death [11, 12]. In this manner, it was clearly demonstrated by molecular joining of doxorubicin to disaccharide, the new molecule doxorubicin disaccharide exhibited better apoptotic effect and fewer side effects in clinical practice [11, 12].

Many of the plant extracts in our previous studies were the basis for identifying new active molecules such as disaccharides, certain microelements and vitamins, which can trigger apoptosis and in higher concentrations (1 mg/mL or more) even dead cells in many cancer cell lines (in the current study in human hepatocellular liver carcinoma cell line HepG2) [13]. It is important to note that the above mentioned molecules showed more or less similar pro-apoptotic effects regardless of the type of cancer cell line used. This was also demonstrated with the molecule docetaxel isolated from the plant *Taxus brevifolia* as described by Huang et al. [14, 15] (https://www.breastcancercare.org.uk/information-support/facing-breast-cancer/going-through-treatment-breast-cancer/chemotherapy/docetaxel-taxotere).

In order to determine the potential anti-cancerogenic effects of the two cold plant extract mixture of *Capsicum chinense* (plant family *Solanaceae*) and *Allium sativum* (plant family *Amaryllidaceae*), we decided to study its natural extracts regarding their potential apoptotic and/or cytotoxic effects on human hepatocellular liver carcinoma cell line HepG2 [13, 16, 17].

## Methods

### Materials

In our research we used the following materials: MTS from Promega (USA), Bradford reagent from Bio-Rad (Germany), z-VAD-fmk, Ac-DEVD-AFC, DTT from Bachem (Switzerland), FBS from Gibco (USA), antibiotics penicillium, streptomycin and glutamax from Gibco (USA), annexin V-PE and 7-AAD for flow cytometry were purchased from BD Biosciences (USA). Fluorescent organelle-specific probe Mitotracker CMXRos was of Molecular Probes (Eugene, OR, USA) and cellobiose, vitamin C, sulphur and selenium were from Calbiochem, Germany. All other reagents (RIPA buffer, caspase buffer, KDMEM, Tryple Select, PBS) were prepared at the institute according to standard procedures described [18, 19].

### Cell lines

The cell line used in the experiments was human hepatocellular liver carcinoma cell line HepG2 acquired from ATCC. Before performing experiments the cells were tested for the cell line identification by isozyme typing and karyotyping. The cells were last tested one month before we started the experiments. Cells were grown in Dulbecco’s modified Eagle’s medium supplemented with 20 % bovine serum (FBS), 1 % penicilium/streptomycin and 1 % glutamax grown to obtain 90 % confluency. When cells were confluent and prepared for experiment with the plant extract, they were transferred in 96-well or 6-well plate, where they were grown in DMEM, free of any kind of supplement of animal origin.

### Preparation of plant extract

*Capsicum chinense* and *Allium sativum* from the plant family *Solanaceae* were grown in a greenhouse. The seeds of above mentioned plants were provided by Sonnentor Kräuterhandels GmbH, Sprögnitz 10, 3910 Zwettl, Österreich, who also made the formal identification of the seeds (*Capsicum chinense* ZO354 AT-BIO-301 and *Alium sativum* ZO325 AT-BIO-301). Once the plants grown from above mentioned seeds in the greenhouse blossomed and ripened into fruits (seeds planted in April, harvest in September), the whole plants were collected and dried. Our calculation was as follows regarding to extrapolation of data in the cited reference and to generally known nutritional value: 100 g of *Alium sativum* contained 1 g cellobiose, 31.2 mg vitamin C, 14.2 μg of selenium and 10 μg of sulphur, while 100 g of *Capsicum chinense* contained 76.4 mg of vitamin C, 8.8 μg of selenium and 36.6 g of cellobiose [20]. Dry plants were ground into a powder. The voucher of the specimen of the powder was not deposited in a publicly available herbarium. The powder of 500 mg was suspended in 50 mL of DMEM to which penicillium and streptomycin were added, together with glutamax (1 % v/v each of antibiotic and glutamax in the final mixture). The mixture was left at room temperature for 3 days and shaken several times in between. After 3 days the extract was filtered and diluted to reach target concentrations (1 μg/mL to 10 mg/mL) and by these means ready for the experiments and HPLC analysis.

### HPLC analysis

We prepared the samples for the HPLC analysis by adding 10 g of the pulverized plant into 10 mL of distilled water. After three days of incubation at room temperature, we filtered the sample through 0.2 μm Sartorius filter for syringes. The filtrate was then mixed with the buffer 2 (1 % TFA (v/v) in 10 % acetonitrile (v/v)) to a final concentration of 10 mg/mL. For the preparation and flushing of the column C4 we used buffer 1 (1 % TFA (v/v) in water).

As a first step we analysed 600 μL of the sample (300 μL of the extract and 300 μL of buffer 2). HPLC analysis duration was 45 min and during this time 31 fractions with different elution times were acquired. The procedure was repeated with a larger sample volume of 1 mL (500 μL of the extract and 500 μL of buffer 2).

Further HPLC analysis was performed in the following manner. The separations were performed using a Finnigan Surveyor HPLC system equipped with a photodiode UV–VIS detector (Thermo Electron Corporation, San Jose, CA, USA) using a 50 mm light-pipe flow cell and data acquisition software ChromQuest version 3.1.6 (Thermo Electron Corporation). The column used was Hypercarb (100 % porous graphitic carbon phase) with dimensions of 100 mm × 3 mm i.d. (Thermo Electron Corporation) and its temperature was kept at 45 °C. UV detection was performed at 300 nm. The flow rate during analysis was constant with 1 mL/min and the injection volume was 10 μL. Mobile phase A consisted of ultrapure water with formic acid 1 % v/v, while mobile phase B consisted of acetonitrile with 1 % v/v formic acid.

Analysis was performed on HPLC instrument Hewlett Packard Series 1100. The instrument was equipped with 1000 μL injection loop, fluorescent detector and ultraviolet detector and integrator. Fluorescence: excitation 280 nm and emission 325 nm, UV detector: 215 nm. As a mobile phase we used one liter of the buffer consisting of 40 % v/v acetonitrile and 60 % v/v distilled water miliQ with the addition of TFA in the final percentage of 1 % v/v. Flow rate was 1.5 mL/min. After the analysis was concluded, we obtained 93 fractions.

### Cell culture

All cells were grown in 10 cm Petri dishes (*N* = 3) to obtain 90 % confluency and afterwards washed with 1 × PBS and treated with Tryple Select 5–10 min. In the next step the cells were transferred into 96-well plate (*N* = 1 for 96-well plate, each concentration in 96 well-plate was represented by *N* = 3 repeats) and 6-well plates (*N* = 1 for 6-well plate, each concentration in 96 well-plate was represented by *N* = 3 repeats) at a ratio of 1 × 10^5^ cells/well (HepG2) and grown overnight. The next day the cells were treated with either the HPLC fraction 10 containing cellobiose sulphur, selenium and vitamin C, solution of pure cellobiose itself or with cellobiose in the combination with added vitamin C, sulphur and selenium in the concentration range of 0.00 l–1000 mg/mL (raising concentrations by a factor of 10; *N* = 3 for each concentration) and incubated for 24 h. The controls are represented by the same cell line (HepG2) maintained in culture in two different ways, with and without antibiotics. All experiments were repeated three times.

### Determination of cell death

#### MTS assay

After the incubation period as described above under the section Cell culture, we estimated cell morphology using light microscopy (Olympus IX71, Japan, magnification 40 and 60). The number of viable cells in proliferation or dead cells were measured by the CellTiter 96^R^ AQ_ueous_ One Solution Reagent, which contains a novel tetrazolium compound. The MTS tetrazolium compound (Owen’s reagent) is bioreduced by cells into a colored formazan product that is soluble in tissure culture medium. Therefore in the end we then added 20 μL of MTS into 100 μL samples in the 96-well plate (*N* = 3 for each concentration). After 45 min of incubation we measured the absorbance at 490 nm with a 96-well plate reader (TECAN XFLUOR4 version V 4.51). The quantity of formazan product as measured by the amount of 490 nm absorbance is directly proportional to the number of living cells in the culture. We calculated the average absorbance of three parallels for control cells, dead cells and individual samples. The percentages of dead cells were calculated according to established formula: (average aborbance of sample - average absorbance of dead cells)/(average absorbance of control cells - average absorbance of dead cells) in percentage. For further analysis of caspase activity in treated HepG2 cells we had chosen the concentrations of plant extracts that induced 20–50 % loss of viability, as determined by the MTS assay (*N* = 3).

#### Caspase (DEVD-ase) activation determination

Cells were cultured and treated as described above. Then, to prepare the cell extracts for caspase activity detection, cells were collected in three parallels, pelleted by centrifugation at 1000 rpm for 5 min and washed twice with PBS. Whole-cell extracts were prepared in 35 μl RIPA buffer (50 mM Tris, pH 8.0, 100 mM NaCl, 0.1 % (w/v) SDS, 1 % (v/v) Nonidet P-40 0.5 % w/v deoxycholic acid, 1 mM EDTA) in three parallels. After 7 min of incubation on ice cells were again subjected to centrifugation this time 5 min on 14,000 rpm. In white 96-well-plate we transferred 40 μl of each samples and added 50 μl of 2 × caspase buffer/ 1 M DTT and 10 μl of DEVD substrate. We gathered 40 μg of protein from cells, untreated and treated with the plant extract in the presence or absence of inhibitor z-VAD-fmk, to determine caspase activity and by measuring the proteolytic cleavage of the fluorogenic substrate Ac-DEVD-AFC (Bachem) as also shown in standard protocols [21]. Before absorbance measurement the plate was incubated at 37 °C in the incubator with 5 % CO_2_. The absorbance was measured by TECAN xfloor4 program at 400–505 nm and DEVD-ase activity calculated in RFU [21].

#### Apoptosis detection by Annexin V and 7-AAD staining

Early apoptotic and dead cells were quantified by flow cytometry measurements of phosphatidylserine exposure and 7-AAD incorporation. For this purpose, the cells were cultured and treated as described in the sections above. Afterwards the cells were washed twice with cold PBS and then resuspended in 1X Binding Buffer at a concentration 1 × 10^6^ cells/mL. Briefly, 100 μL aliquots of cells were labelled with 5 μl annexin V-PE and 5 μl 7-AAD according to the manufacturer’s instructions and transferred into 5 mL tube for each concentration of plant extract, pure cellobiose or combination of molecules separately. The cells were gently vortexed and incubated for 15 min at room temperature (25 °C) in the dark. In the end 400 μl of 1X binding buffer was added to each tube. The cells were then subjected to flow cytometry analysis using FACScalibur flow cytometer (BD Biosciences) and analyzed with the CellQuest software within one hour.

#### Determination of apoptotic pathway

To determine by which apoptotic pathway (intrinsic, extrinsic) the plant extract triggers apoptosis we used Mitotracker Red CMXRos to assess and monitor the integrity of mitochondria. Cells were cultured and treated as previously described, and after the incubation period, cells were washed twice with cold PBS and then resuspended in Mitotracker CMXRos, which was added to the cells at a final concentration of 20 nM. Following 30 min incubation at 37 °C the cells were washed in PBS and again re-suspended in PBS for the measurement on a flow cytometer. Afterwards we measured red fluorescence of 5000 or 10,000 cells per sample, corresponding to mitochondria using the FL3 channel on a flow cytometer.

### Mass spectrometric analysis

Mass spectrometric analysis measurements of the *Solanaceae* plant extract were run on a hybrid quadrupole time of flight mass spectrometer (Q-TOF) provided with an orthogonal Z-spray ESI interface (Waters Micromass, Manchester, UK). Mass spectrometer was interfaced to an ultra performance liquid chromatography (UPLC) system based on a Waters Acquity (Waters, Milford, USA) binary pump with a BEH C18 column (1.7 μm, 50 × 2.1 mm i.d). The mobile phases consisted of water and acetonitrile with a mixture of 0.1 % of formic acid in water. Compressed nitrogen (99.99 %, Messer Slovenia) was used as both the drying and the nebulising gas. The nebulizer gas flow rate was set to approximately 20 L/h and the desolvation gas flow rate to 600 L/h. A cone voltage of 30 V and a capillary voltage of 2.7 kV were used in a positive ion mode. The desolvation temperature was set to 250 °C and the source temperature to 150 °C. The mass resolution of approximately 9500 (fwhm) was used for determination of elemental composition with TOF mass spectrometer. MS and MS/MS spectra were acquired in centroid mode over an m/z range of 50–1000 in a scan time of 0.25 s and inter scan time of 0.05 s. For MS/MS experiments, argon (99.995 %, Messer Slovenia) was used as collision gas at a pressure of approximately 2 × 10^5^ mbar in the collision cell. Product ion spectra were generated at collision energies profile: 10–30 V. The detector potential was set to 2300 V. Reproducible and accurate mass measurements at 10,000 mass resolution were obtained using an electrospray dual sprayer with leucine encephalin ([MH] + = 556.2771) as a reference compound, introduced into the mass spectrometer alternating with a sample solution. The data station operating software was Mass Lynx v. 4.1 (Micromass, Manchester). Several chromatographic peaks were identified with mass spectrometric detection.

### NMR analysis

^1^H-^1^H and ^1^H-^13^C correlation NMR spectra of plant extract were acquired on a Agilent (Varian) VNMRS 800 MHz NMR spectrometer equipped with a cold probe. ^1^H-^1^H TOCSY spectra were recorded with mixing times of 20, 40, 60 and 80 ms on an Agilent (Varian) Unity Inova 300 MHz NMR spectrometer equipped with ID probe. All data were recorded in ^2^H_2_O at 298 K. ^1^H chemical shifts were referenced to the residual solvent signal of ^2^H_2_O at δ 4.80 ppm. ^13^C chemical shifts have been determined on the basis of ^1^H-^13^C correlations in HSQC spectrum and are reported relative to TMS (δ = 0 ppm).

### Analysis and statistics

The percentage of apoptotic and dead cells were reported as mean ± standard error. Data were analyzed using the two-way analysis of variance (ANOVA). Differences between experimental and control groups and among different exposure groups were regarded as statistically significant at the level of * *p* < 0.05, **p < 0.001 and *** *p* < 0.0001.

## Results

### Isolation of the fraction with active molecules

**Fig. 1:**
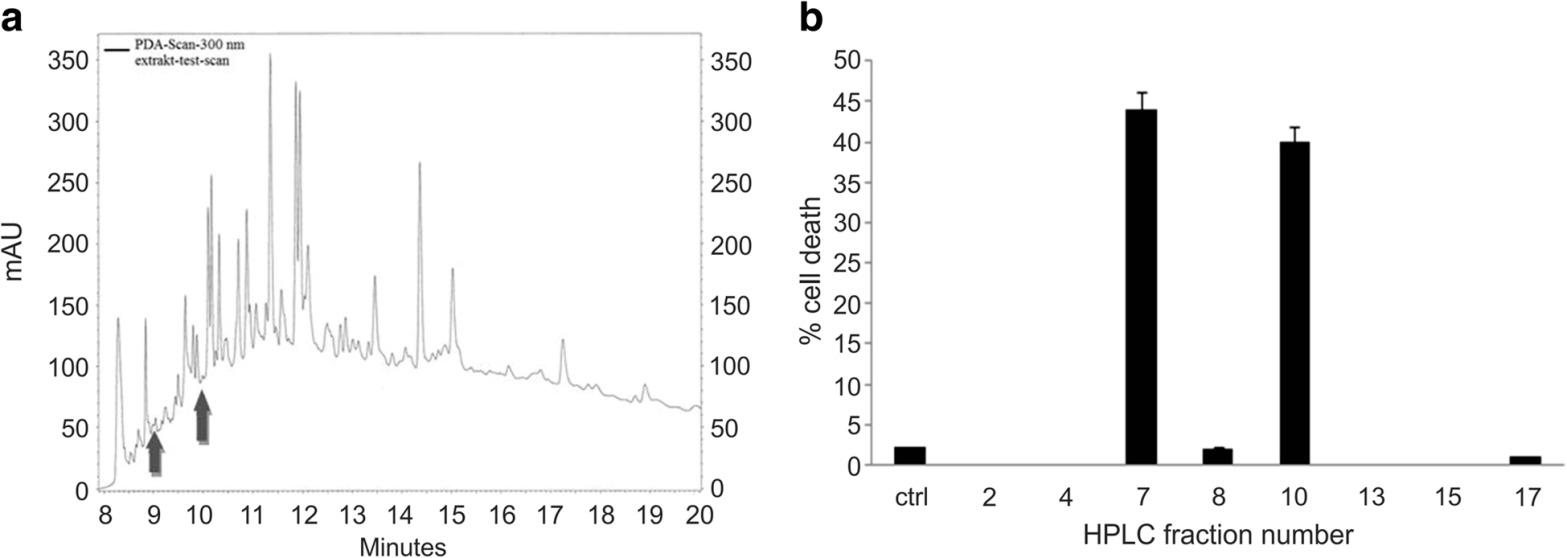
HPLC analysis with hypercarb column and analysis of fractions by MTS test. **a** The fractions with active ingredients of the plant extract were eluted after 9.3 min and 10 min (see *arrows*). **b** Quantification of cell death measured by MTS test after treating the HepG2 cells with the HPLC samples. The fraction number 7 represents the first fraction with active ingredients eluted after 9.3 min and the fraction number 10 the second fraction with active ingredients eluted after 10 min

Whole plants Capsicum chinense and Allium sativum were individually ground and the extracts performed as described in Materials & Methods. The extracts of both plants were then mixed together and used further for HPLC analysis When performing HPLC analysis of both cold whole plant extracts mixture of *Capsicum chinense* and *Allium sativum*, we acquired 93 fractions (Fig. 1a). The fractions of the mixture of both cold plant extracts of *Capsicum chinense* and *Allium sativum* together acquired by HPLC analysis were analysed by MTS test to determine, which fraction triggers loss of viability of cancer cells and has consequently the crucial effective molecules, we were searching for. Using MTS assay, we determined greatest loss in viability of cancer cells when these were treated with the fractions of elution time of 9.3 and 10 min (Fig. 1b). The rate of cell death in untreated cells was 2,1 % as also shown in the graph (Fig. 1b). The fraction with the elution time 9.3 corresponds to a sample number 7 (designated from here on as fraction 7) and the one with the elution time 10 min to a sample number 10 (designated from here on as fraction 10). For most of our further experiments (mass spectrometric analysis and NMR analysis) we used only HPLC fraction 10, since fraction 7 was unstable and its pro-apoptotic effect was lost 24 h after it was eluted from the column.

### Characterisation of active molecules

**Fig. 2:**
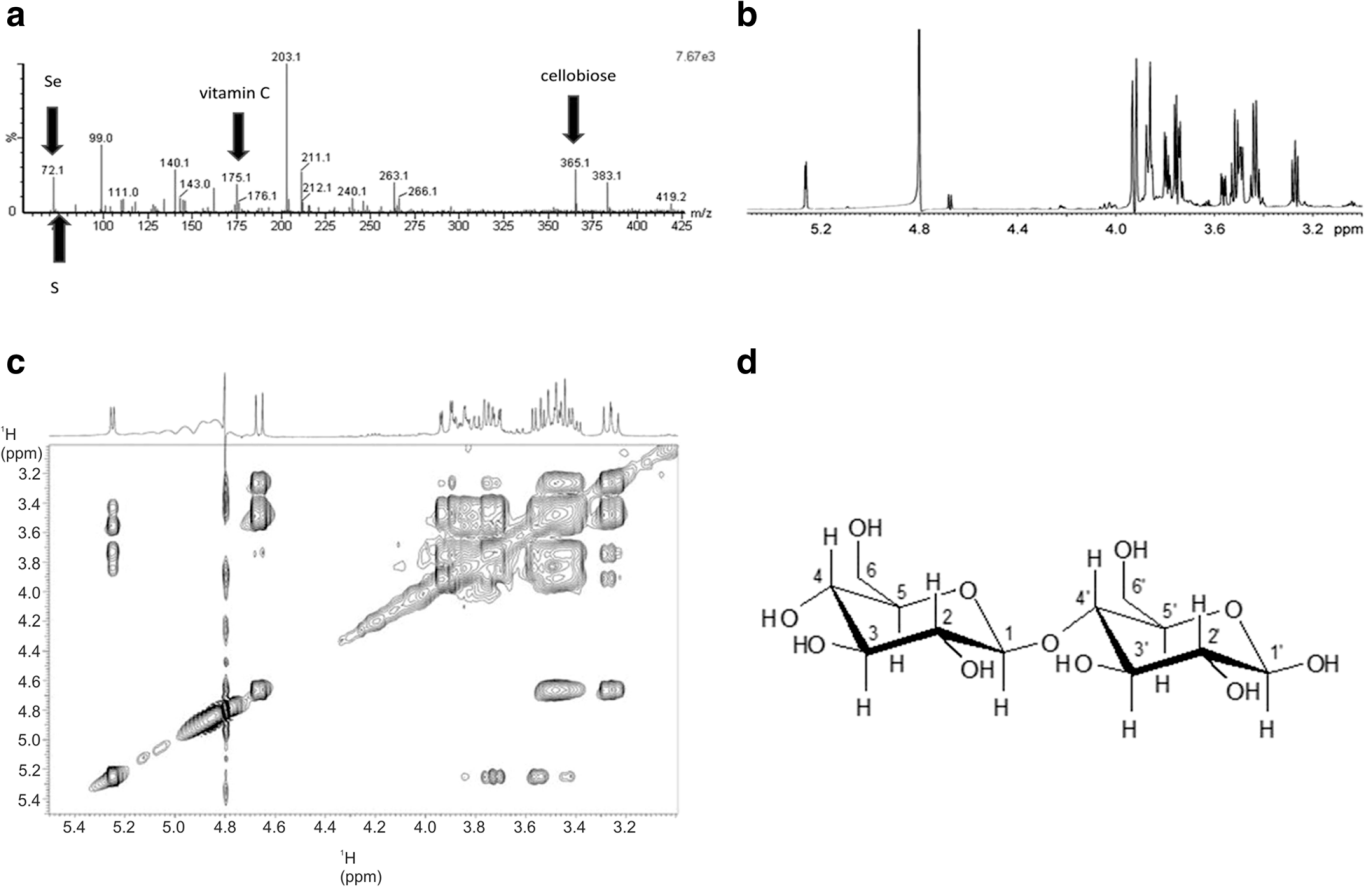
Mass spectrometric and NMR analysis of the active fraction 10. **a** Mass spectrometric analysis of the HPLC fraction 10. The active fraction contained several molecules. Peak 383.1 shows the cellobiose, peak 176.1 shows vitamin C, peak 72.1 shows selenium and small peak after selenium shows sulphur, which was present in extremely low quantities. Other peaks represent different monosaccharides and disaccharides. **b** NMR signals showing rich composition of HPLC fraction 10 with leading quantity of cellobiose. **c** NMR analysis of the HPLC fraction 10 showing the main composition to be cellobiose. **d** Structure of cellobiose

Mass spectrometric analysis and NMR analysis were performed in order to further explore, whether the selected fraction 10 contains only one active molecule or more (Fig. 2a).

Mass spectrometric analysis of the fraction 10 showed the peak 383.1 with molecular weight of 342.3 g/mol what is the mass of cellobiose, what was in further experiments also confirmed with the NMR analysis. There were also several other peaks shown peak 176.1 shows vitamin C, peak 72.1 shows selenium and small peak after selenium shows sulphur, which was present in extremely low quantities. Other peaks represent different monosaccharides and disaccharides (Fig. 2a).

In the next step we performed the NMR analysis in order to confirm the data acquired with the mass spectrometric analysis.

The ^1^H and ^13^C resonances were assigned through analysis of their respective multiplicities and integrals in proton spectrum as well as through-bond connectivity in homo- and heteronuclear 2D spectra. Two anomeric protons were clearly resolved at 4.67 and 5.26 ppm (Fig. 2b).

Variation of mixing time in TOCSY experiment allowed assignment of individual protons. The entry point for the assignment of proton resonances belonging to the second monosaccharide unit was anomeric proton H1' at δ 5.26 ppm, which enabled unambiguous assignment of H2'-H6' proton signals (Table 1).

**Table 1:** ^1^H NMR chemical shifts (in ppm) and coupling constants (in Hz) for fraction with cellobiose^a^

Proton	*δ*	Coupled protons	*J*	Proton	*δ*	Coupled protons	*J*
H1	4.67	H1-H2	8.3	H1'	5.26	H1'-H2'	4.1
H2	3.27	H2-H3	9.6	H2'	3.48	H2'-H3'	9.6
H3	3.52	H3-H4	8.9	H3'	3.74	H3'-H4'	7.6
H4	3.43	H4-H5	9.6	H4'	3.44	H4'-H5'	9.6
H5	3.49	H5-H6a	6.2	H5'	3.86	H5'-H6'a	5.5
		H5-H6b	2.1			H5'-H6'b	2.1
H6a	3.75	H6a-H6b	12.4	H6a'	3.79	H6'a-H6'b	12.4
H6b	3.93	H6b-H6a	12.4	H6b'	3.87	H6'b-H6'a	12.4

^a^Reported chemical shifts correspond to NMR spectra acquired in ^2^H_2_O at 298 K. The complete set of ^1^H and ^13^C NMR chemical shifts is available in Materials and methods: NMR analysis section

Trans orientations of carbohydrate protons from H2 to H5 as well as from H2' to H5' were established on the basis of vicinal ^1^H–^1^H coupling constants (Table 1), which suggested that both monosaccharide units correspond to glucose. ^1^H NMR chemical shifts of cellobiose are given in Table 1, with the corresponding atom numbering shown in Fig. 2c. ^3^ *J*_H1H2_ coupling constant of 8.3 Hz indicated trans orientation of H1 with respect to H2, which furthermore suggested β-glycoside bond in the disaccharide (Fig. 2c).

The ^1^H NMR spectrum showed signals in the region between 3.3 and 5.3 ppm, which corresponds to cellobiose (Fig. 2d), which was present in the fraction in majority in contrast to vitamin C, sulphur in selenium, where concentrations were lower.

### Effect of HPLC fraction 10 on HepG2 cells

**Fig. 3:**
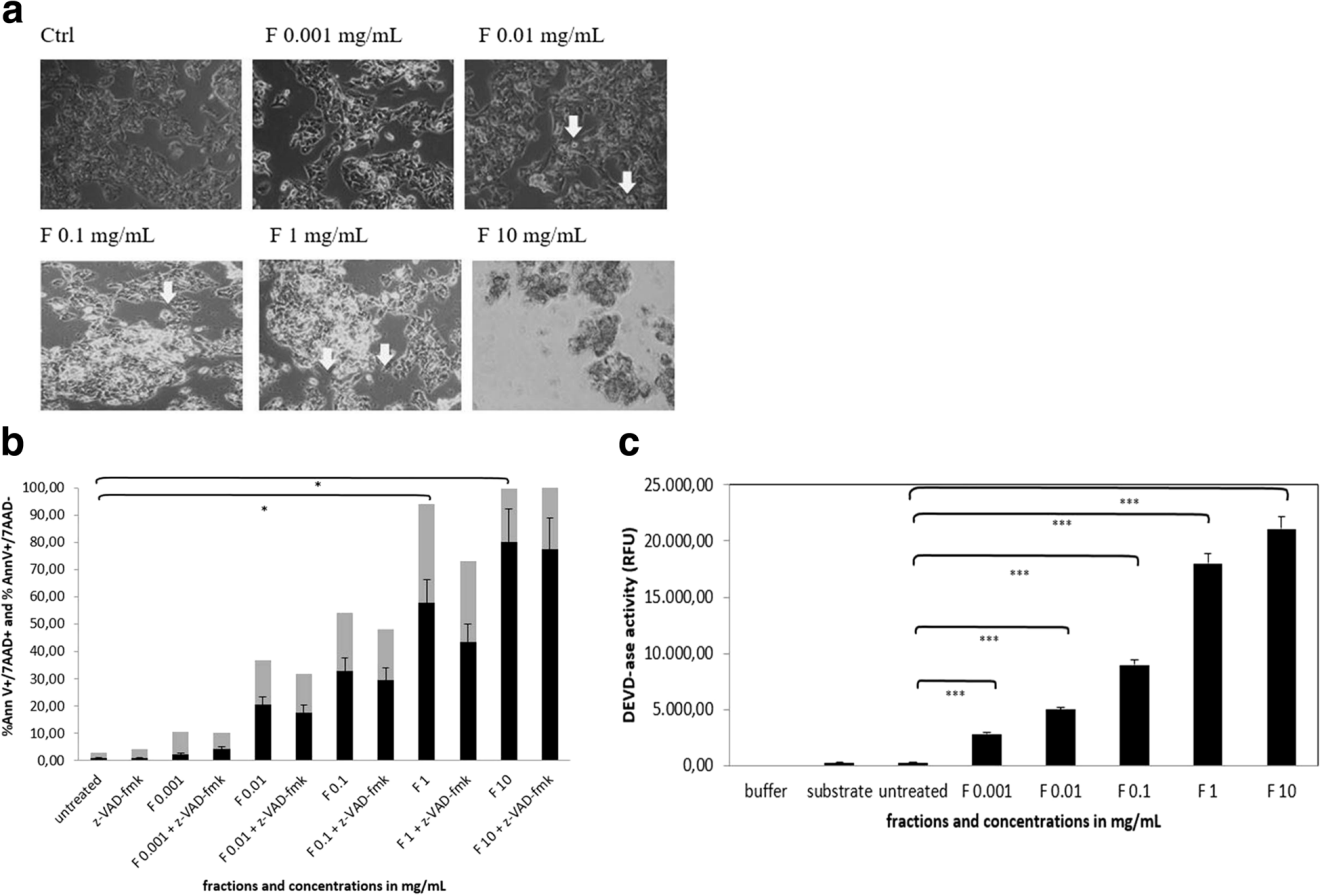
Effect of the HPLC fraction 10 on cell morphology, viability and activation of caspase-3. **a** Effect of the HPLC fraction 10 on triggering cytotoxic insult in HepG2 cancer cells after 3 h of incubation in the concentration range 0.001–10 mg/mL. Note apoptotic cells with different concentrations (see *arrows*). **b** Viability of HepG2 cells measured by FACS after treating them in the concentration range 0.001–10 mg/mL with the HPLC fraction 10. Note that for all experiment the second stable fraction eluted after 10 min was used. Effect on cells pretreated with z-VAD-fmk was measured as well. *Grey bars* are representing the percentage of apoptotic cells and *black bars* percentage of dead cells. Error bars denote mean ± SEM. *p < 0.05; ** *p* < 0.001; *** *p* < 0.0001. **c** DEVD-ase activity (caspase −3 activity) in HepG2 cells after the treatment of the cells with HPLC fraction 10 in the concentration range 0.001–10 mg/mL. DEVD-ase activity measured in arbitrary units (RFU). Error bars denote mean ± SEM. *p < 0.05; ** *p* < 0.001; *** *p* < 0.0001

As shown in Fig. 3a, the morphology of the cells suffering from a cytotoxic insult of fraction 10 can be readily observed by light microscopy. Cells treated in the concentration range of 0.01–1 mg/mL exhibited typical morphology of a cytotoxic insult, including cell shrinkage and in some cases, detachment from the surface (shown with arrows).

The pro-apoptotic effect of isolated plant extract on HepG2 cancer cells was determined using flow cytometry via Annexin V FITC and 7-AAD, where fraction 10 triggered 23,77 % ± 4,38 apoptotic cells of HepG2 cancer cells (*N* = 3 for each samples and concentration) (Fig. 3b). Optimal response was obtained with concentration range 0.01–1 mg/mL. It is visible from the analysis, that we obtained 20,40 % ± 11,17 dead cells (Fig. 3b, column F 0.01, black bar), and 16,40 % ± 2,60 early apoptotic cells in HepG2 cultures (Fig. 3b, column F 0.01, grey bar), which was enough to confirm the onset of apoptosis. At the same time the DEVD-ase activity was measured in order to also determine whether apoptosis can be blocked by z-VAD-fmk (Fig. 3b; *N* = 3 parallels and repeats). At higher concentrations, determination of cell death by flow cytometry revealed that 100 % of cells underwent the apoptotic process, namely when treated with 1 to 10 mg/mL of fraction 10 (*N* = 3 parallels and repeats). In contrast to the plant extract only, the effect of fraction 10 was already present after 3 h of the treatment of the cancer cells (data not shown) [11].

The highest activation of caspase-3 (DEVD-ase activity), which was 21.000 RFU, was achieved when treating cancer cells with 10 mg/mL concentration (*N* = 3 parallels and repeats, *p* < 0.0001) for 3 h, while 0.01 mg/mL concentration (*N* = 3 parallels and repeats) produced only weak activation (5.000 RFU; *p* < 0.0001) of the enzyme in the same time span (Fig. 3c).

### Effects of cellobiose solution on HepG2 cells

**Fig. 4:**
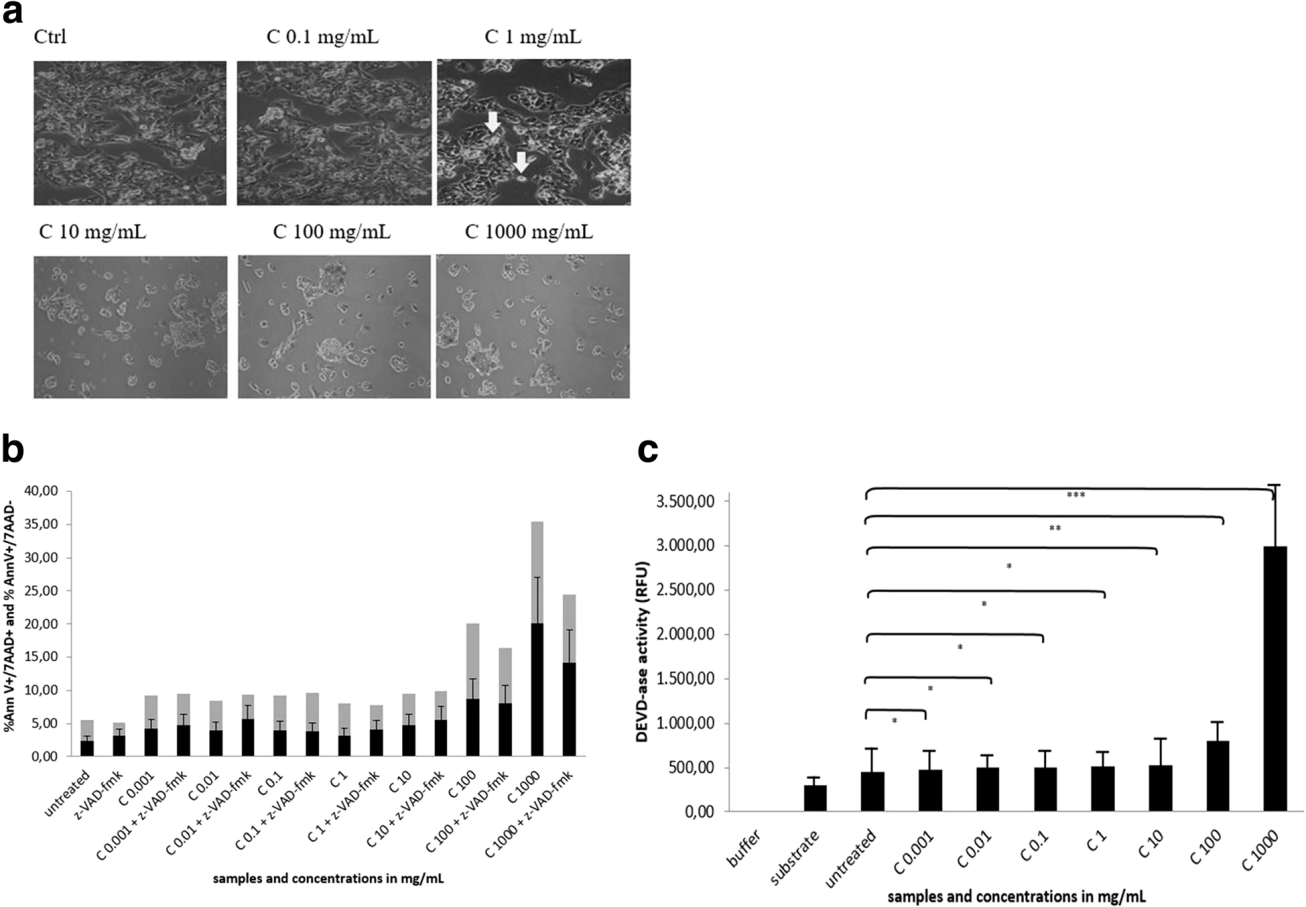
Effect of solution of pure cellobiose on HepG2 cell morphology, viability and activation of caspase-3. **a** Effect of solution with pure cellobiose on HepG2 cancer cells after 24 h of incubation in the concentration range 0.1–1000 mg/mL. Note apoptotic cells with different concentrations (see *arrows*). **b** FACS analysis of viability of HepG2 cells after being treated with the solution of pure cellobiose in the concentration range 0.001–10 mg /mL after 24 h. Effect on cells pretreated with z-VAD-fmk was measured as well. *Grey bars* are representing the percentage of apoptotic cells and *black bars* percentage of dead cells. Error bars denote mean ± SEM. *p < 0.05; ** *p* < 0.001; *** *p* < 0.0001. **c** Caspase activity in HepG2 cells after the cells were being treated with different concentrations of pure solution of cellobiose in the concentration range 0.001–1000 mg/mL after 24 h. Error bars denote mean ± SEM. *p < 0.05; ** *p* < 0.001; *** *p* < 0.0001

For comparison, we set out to determine the effect of pure cellobiose itself on HepG2 cells. We have shown that pure cellobiose induces cell death of HepG2 within 24 h when applied at 100 to 1000 mg/mL, as determined by morphological assessment. It was observed that with higher concentrations, such as 10–1000 mg/mL, the cells (*N* = 3 parallels and repeats) were not confluent any longer and were bursting in the process (Fig. 4a).

This was further confirmed with flow cytometry, where we discovered that a concentration of 100 mg/mL induces cell death in 20,10 % ± 6,97 (not statistically significant), while 1000 mg/mL of cellobiose induces cell death in 35,50 % ± 8,27 of HepG2 (Fig. 4b). However, the effect of pure cellobiose was visible only after 24 h with fewer apoptotic cells (5,00 % ± 3,41) in concentrations below 100 mg/mL, which was not statistically significant (*N* = 3 parallels and repeats) as shown in Fig. 4b.

Pretreatment of cells with z-VAD-fmk did not rescue cells from cell death within 24 h. This was comparable with the caspase activation in the same time span (Fig. 4c), where the cells were bursting in the process.

### Effects of unique cellobiose complex with vitamin C, sulphur and selenium on HepG2 cells

**Fig. 5:**
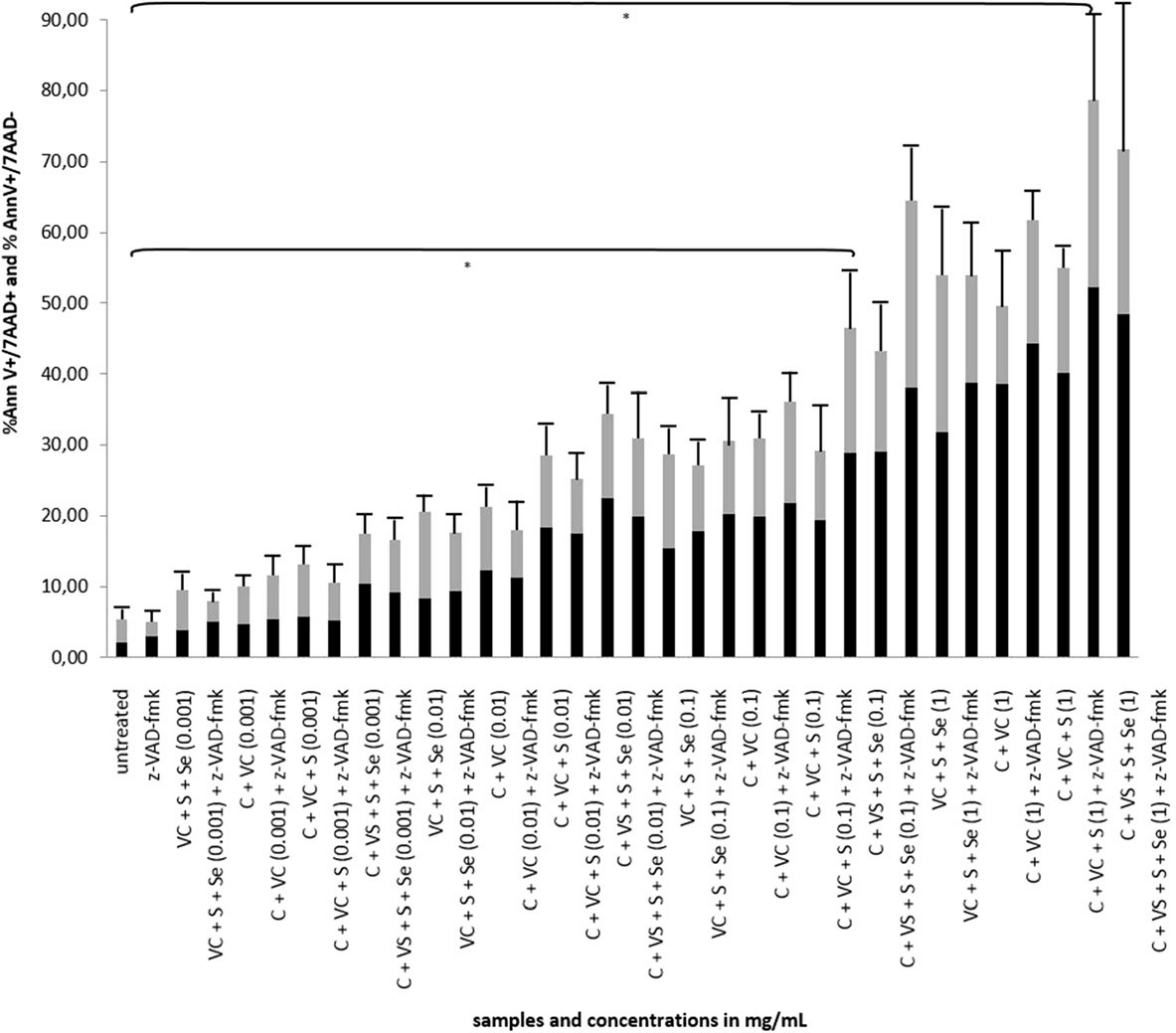
Effect of combination cellobiose/S/Se/vitamin C on HepG2, viability. Flow cytometry analysis and number of apoptotic and dead cells in HepG2 cancer cell line after treatment with unique cellobiose complex with vitamin C, sulphur and selenium (marked as C + VC + S + Se) together with the treatment of individual solutions of cellobiose (marked as C), vitamin C (marked as VC), sulphur (marked as S) and selenium (marked as Se) together with the possible blockade of DEVD-ase activity by z-VAD-fmk in the concentration range 0.001–1 mg/mL after 24 h. *Grey bars* are representing the percentage of apoptotic cells and *black bars* percentage of dead cells. Error bars denote mean ± SEM. *p < 0.05; ** *p* < 0.001; *** *p* < 0.0001

Since we have discovered pure cellobiose itself is not able to strongly induce apoptosis and cell death, we repeated experiments with cellobiose complex according to mass spectrometric analysis. For this purpose we performed multiple experiments in various combinations with cellobiose (cellobiose/vitamin C, cellobiose/vitamin C/sulphur and cellobiose/vitamin C/sulphur/selenium). We discovered that the combination of cellobiose, vitamin C, sulphur and selenium (cellobiose/C/S/Se) produced comparable results as HPLC fraction 10, as seen in FACS analysis of viability (Fig. 5). This final cocktail with all ingredients induced even better results than the HPLC fraction 10, since the HPLC fraction 10 induced 27,10 % ± 7,88 (*p* < 0.05) of apoptotic/dead cells in concentration of 0.1 mg/ml (Fig. 3b) in contrast to cellobiose/C/S/Se complex, where there were 46,60 % ± 7,73 (*p* < 0.05) dead cells.

**Fig. 6:**
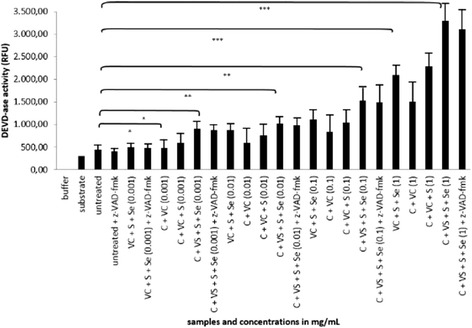
Effect of combination cellobiose/S/Se/vitamin C on activation of caspase-3 in HepG2. DEVD-ase activity after the treatment of the HepG2 cells with the solutions of unique cellobiose complex with vitamin C, sulphur and selenium (marked as C + VC + S + Se) together with the treatment of individual solutions of cellobiose (marked as C), vitamin C (marked as VC), sulphur (marked as S) and selenium (marked as Se) in the concentration range from 0.001 to 1 mg/mL after 24 h. DEVD-ase activity was measured in arbitrary units (RFU). Error bars denote mean ± SEM. *p < 0.05; ** *p* < 0.001; *** *p* < 0.0001

We have also observed that pre-treatment of cells with z-VAD-fmk did not rescue cells from apoptosis and dead cells suggesting there must be also non-caspase dependent apoptotic pathway involved (Fig. 6).

**Fig. 7:**
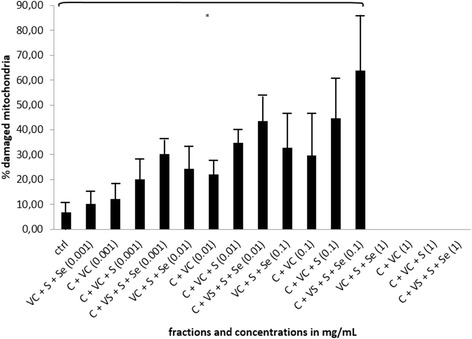
Effect of combination cellobiose/S/Se/vitamin C on integrity of mitochondria in HepG2. Analysis of the integrity of mitochondria with MitoTracker RedCMXros in HepG2 cancer cells after the treatment with the unique cellobiose complex with vitamin C, sulphur and selenium (marked as C + VC + S + Se) together with the treatment of individual solutions of cellobiose (marked as C), vitamin C (marked as VC), sulphur (marked as S) and selenium (marked as Se) in the concentration range 0.001–1 mg/mL after 24 h. Error bars denote mean ± SEM. *p < 0.05; ** *p* < 0.001; *** *p* < 0.0001

The process of apoptosis led to loss of mitochondrial integrity, wherein unique cellobiose/C/S/Se complex with the concentration of 0.1 mg/ml resulted in 63,90 % ± 22,83 (*p* < 0.005) of damaged mitochondria, while higher concentration induced bursting of mitochondria, where its debris could not be detected anymore (Fig. 7).

## Discussion

In recent years, immense interest has been generated for saccharides especially for d-allose, monosaccharides and disaccharides based on scientific rationale from previous studies which have shown that sugars are in some cases pro-apoptotic in variety of cancer cells [22, 23] (http://www.canceractive.com/cancer-active-page-link.aspx?n=3352). For the present study we have focused on the effect of the mixture of two cold plant extracts from *Allium sativum* from the plant family *Alliaceae* and *Capsicum chinense* from the plant family *Solanaceae* in comparison to pure synthetic cellobiose or to solution of cellobiose combined with vitamin C, sulphur and selenium.

Agents that can modulate apoptosis may be able to affect steady - state cell population, which may be useful in cancer therapy [24, 25]. As we have shown previously the mixture of two cold plant extracts from *Alium sativum* and *Capsicum chinense* triggered the onset of apoptosis and cell death in cancer cell line HepG2 [13]. In the current context of mixture of two cold plant extracts mentioned above, our aim was to determine which molecules in the plant extract are crucial for the onset of apoptosis and cell death. For this purpose we analysed the plant extract mixture with the HPLC analysis using the hypercarb column (Fig. 1a). The results showed the rich composition of the plant extract mixture. In a subsequent MTS assay we identified that from all 93 fractions, only one stable fraction (designated as fraction 10) was capable of significantly inducing apoptosis and cell death in HepG2 cells (Fig. 1b). By mass spectroscopic analysis (Fig. 2a) we identified a number of peaks including cellobiose (peak 383.1), vitamin C (peak 176.1), selenium (peak 72.1) and sulphur (small peak after selenium), what was also confirmed by NMR analysis (Fig. 2b–d).

According to our previous results, there is a difference between response of HepG2 cancer cell line to the plant extract mixture or to individual molecules present in this plant extract in terms of both the time of onset and extent of apoptosis induction [13]. This was clearly seen in experiments for the identification of rate of apoptosis triggered by plant extract mixture in contrast to individual molecules from the plant extract as also shown by other research groups [26–30]. We demonstrate in this study that when using HPLC fraction 10 containing cellobiose, selenium, sulphur and vitamin C, already at 0.01 mg/ml, it induces 20 % of dead cells and 16 % of apoptotic cells within the first 3 h of treatment (Fig. 3a and b). By raising the concentration of HPLC fraction 10, the pro-apoptotic effect increases in a dose-dependent manner, reaching plateau at 10 mg/ml, where 100 % cells in the culture are dead (Fig. 3b). This could not be achieved using cellobiose alone, where 10.000-fold higher concentrations were required to induce comparable results. Furthermore, cellobiose only induced similar results after a much longer, 24 - h incubation period (vs 3 h for HPLC fraction 10) (Fig. 4a and b). In this manner, our results are similar to those of Yang and Naha, who have similarly studied the effect of glucose and suggested that very high dosages of sugars are necessary to induce programmed cell death [7, 8].

Considering mechanisms of induction of cell death by our plant extract mixture, we tested the capacity of z-VAD-fmk, a pan-caspase inhibitor, to decrease the percentage of apoptotic/dead cells treated with HPLC fraction 10. As shown in Fig. 3b, preincubation of cells with z-VAD-fmk slightly decreased apoptosis induction, however, these results were not significant. Similar effect of caspase inhibition was also seen when we used pure cellobiose or the unique complex consisting of cellobiose, vitamin C, selenium and sulphur (cellobiose/C/Se/S) (Figs. 4b, 5b). These results suggest that the HPLC fraction 10 and cellobiose itself trigger PCD and possibly another apoptotic pathway that is not entirely caspase-dependent. A similar observation was made by Bhushan and Swayamjot and their research group, who demonstrated that plant-derived polyphenol- and triterpenediol-induced apoptosis of cancer cells is not triggered by a single pathway [5, 14]. Similar results were obtained by Lamy et al. [31]. However, we discovered that caspase-3 was strongly activated in HepG2 cells throughout the whole concentration range of HPLC fraction 10 (from 0.001 to 10 mg/mL), but only at 1000 mg/ml in the case of pure cellobiose itself (Figs. 3c and 4c). Therefore considering cellobiose itself, our results clearly show that in lower concentrations it is not capable of inducing PCD. This could be partly due to the fact that cellobiose is degraded to glucose which further decreases its effectiveness. We can compare these results with a study published by Yang et al. and Lorenzi et al., where they observed cytotoxicity of high concentration of saccharides, especially glucose, to cultured vascular endothelial cells [8, 32].

According to our previous studies on cancer cell line HepG2, we decided to examine the pro-apoptotic capacity of a unique complex where cellobiose is supplemented in small amounts with vitamin C, sulphur and selenium, in similar ratios as observed in HPLC fraction 10 [13, 33]. This combination proved to be optimal in triggering apoptosis in cancer cells, including in HepG2 cell line. We can speculate what happens to cellobiose once in water environment. Disaccharides tend to have different stereochemical properties in different environments. Cellobiose tends to be in packed conformation when not in water. This is due to its nature of 1-4βbond, which enables it to be more stable in combination with elemements such as selenium, sulphur, manganese and other (in our case this can also be vitamin C). By these means the apoptotic effect of this combination of molecules is retained, while cellobiose in water forms more linear conformation and it is therefore more susceptible to degradation and its apoptotic effect is quickly lost [34]. We have observed the loss of viability (Fig. 5), when HepG2 cells were treated with cellobiose complex for 24 h, where concentrations from 0.01 mg/mL onwards induced from 22 to 52 % of dead cells and from 12 to 26 % of apoptotic cells, which represented a significant increase compared to controls. The findings were in correlation with caspase-3 activation, which was dose-dependent, with the highest activation of caspase-3 observed also with the highest concentration of 1 mg/mL of cellobiose complex (Fig. 6). Furthermore, the significant damage of mitochondria, where more than 60 % of mitochondria lost their integrity, occurred with the cellobiose complex concetration of 0.1 mg/mL, suggesting the cellobiose complex triggers apoptosis also partly through intrinsic apoptotic pathway. Mitochondira integrity at 1 mg/ml could not be detected, since the mitochondria simply bursted and were undetectable. This also suggests the involvement of necrotic cell death besides activation of intrinsic apoptotic pathway, which was also shown by Yang, Zhang, Gurfinkel and Bakshi in case of cytotoxicity by high concentration of saccharides and other natural based molecules [8, 28, 35, 36].

In summary, our results suggest that the rich composition of plant extract mixture, which was subsequently defined as a complex of cellobiose, sulphur, selenium and vitamin C, is the combination, which extensively amplifies apoptosis in cancer cells. The most commonly used medicines are administered in combination therapies, which is neccessary in treating many types of cancer. These combinations are often toxic to normal cells and have severe side effects, including resistance treatment, disease relapse and often requiring prolonged or life time treatment [1, 4, 11]. In this context, the combination treatment of safe natural substances including those found in our study, holds a promise as a potential new treatment of patients with liver cancer.

## Abbreviations

7-AAD: 7-Aminoactinomycin D
Ac-DEVD-AFC: Acetyl-Asp-Glu-Val-Asp-7-amino-4-trifluoromethylcoumarin
DMEM: Dulbecco’s modified Eagle’s medium
DTT: Dithiothreitol
EDTA: Ethylene diamine tetra-acetic acid
FBS: Fetal bovine serum
FITC: Fluoroscein isothiocyanate
HepG2: Hepatocellular carcinoma G2 cell line
HPLC: High Performance Liquid Chromatography
MS: Mass spectrometry
MTS: 3-(4,5-dimethylthiazol-2-yl)-5-(3-carboxymethoxyphenyl)-2-(4-sulfophenyl)-2H-tetrazolium
NCI: National Cancer Institute
NMR: Nuclear magnetic resonance
PBS: Phosphate buffered saline
PCD: Programmed cell death
PE: Phycoerythrin
RFU: Relative fluorescence units
RIPA: Radioimmunoprecipitation assay buffer
ROS: Reactive oxygen species
USDA: United States Department of Agriculture
z-VAD-fmk: N-benzyloxycarbonyl-Val-Ala Asp(Ome)fluoromethylketone
